# Multiplexed-tandem PCR (MT-PCR) assay to detect and differentiate gastrointestinal nematodes of alpacas

**DOI:** 10.1186/s13071-018-2963-9

**Published:** 2018-06-28

**Authors:** Mohammed H. Rashid, Hagos Gebrekidan, Abdul Jabbar

**Affiliations:** Department of Veterinary Biosciences, Melbourne Veterinary School, Faculty of Veterinary and Agricultural Sciences, The University of Melbourne, Werribee, Victoria Australia

**Keywords:** South American camelids, Alpacas, Llamas, Nematodes, PCR, Australia

## Abstract

**Background:**

Gastrointestinal nematodes (GINs) frequently infect South American camelids (alpacas and llamas) and cause economic losses due to reduced production of fiber, meat and/or leather. Our knowledge about the epidemiology and diagnosis of GINs in llamas and alpacas is limited, and reliable keys for the identification of the third-stage larvae (L3s) of some common nematodes (such as *Camelostrogylus mentulatus*) that infect alpacas and llamas remain undescribed. In this study, we modified two existing semi-quantitative multiplexed-tandem (MT)-PCR assays, originally developed for the GINs of sheep and cattle, to reliably detect and differentiate the common genera/species of GINs in the faeces of alpacas.

**Results:**

Following the establishment of the MT-PCR assay using positive and negative control samples, alpaca faecal samples were tested to validate the assay to detect and differentiate nematode genera/species, including *C*. *mentulatus*, *Cooperia* spp., *Haemonchus* spp., *Oesophagostomum* spp., *Ostertagia ostertagi*, *Teladorsagia circumcincta* and *Trichostrongylus* spp. Sequencing of the MT-PCR products demonstrated specific (100%) amplification of the target nematode genera/species. Additionally, a comparison of results of the MT-PCR assay and the morphological identification of adult worms collected from the same 35 alpacas revealed that there was a good agreement (37–94%) between the two methods. However, some discrepancies were observed between the results of the MT-PCR assay and the morphological identification of adult worms.

**Conclusions:**

The MT-PCR platform is an accurate, sensitive and rapid method for the diagnosis of GINs in alpacas, and it can be used as a substitute to larval culture to identify common nematodes in the faeces of alpacas and llamas.

## Background

A variety of gastrointestinal nematodes (GINs) can cause parasitic gastroenteritis in South American camelids (SACs), alpacas (*Lama pacos*) and llamas (*Lama glama*). Some of these GINs are host-specific (e.g. *Graphinema auchenia* and *Lamanema chavezi*) and occur in native habitats (i.e. South America) of alpacas and llamas, while others are shared between SACs and domestic ruminants (e.g. *Cooperia* spp., *Haemonchus contortus*, *Nematodirus* spp., *Oesophagostomum* spp., *Ostertagia ostertagi*, *Teladorsagia circumcincta* and *Trichostrongylus* spp.) [1–3]. These nematodes can lead to considerable morbidity and even death in SACs, leading to significant economic losses [4, 5]. Farmers frequently use various classes of anthelmintics to control parasitic gastroenteritis in alpacas and llamas [2, 3], although no anthelmintic is registered against GINs in SACs. The under-dosing of anthelmintics could potentially lead to the development of resistance in GINs of alpacas and llamas [3, 6, 7] as it has been reported in those of small ruminants [8].

Although GINs of SACs have been the subject of intermittent studies over the past 25 years, our knowledge on the epidemiology and control GINs in SACs is still limited [3]. For instance, like domestic ruminants, the diagnosis of GINs in SACs is based on faecal egg counts (FEC) and nematode larval culture (LC). However, these tests are laborious and have low sensitivity and specificity. In addition, the LC requires experienced personnel for accurate identification of the third-stage larvae (L3s) as many nematode species are difficult to distinguish morphologically [9]. Furthermore, keys for the identification of L3s of some GINs that commonly infect SACs (e.g. *C. mentulatus*) remain undescribed. To overcome these challenges, molecular diagnostic tools can be used to accurately identify GINs of SACs like those of domestic ruminants [10]. For instance, the multiplexed-tandem PCR (MT-PCR) assay is a type of real-time PCR method that uses several primer pairs for the detection of multiple pathogens in one sample [11]. This assay consists of two amplification steps where the primary amplification involves a ‘target enrichment’ using outer primer sets with a small number of PCR cycles, whereas the secondary amplification ‘quantification step’ utilises target-specific, nested or inner primers to target a region within the product from the primary amplification [11]. To date, MT-PCR has been applied for simultaneous detection of a number of pathogens of veterinary and medical significance, including fungi [12], enteric pathogens of humans [13], GINs of sheep [14, 15] and cattle [16], and toxigenic cyanobacteria [17].

A recent study in Australia showed that alpacas can be infected with a variety of GINs which also infect sheep and cattle. Furthermore, a high prevalence of a stomach worm, *C. mentulatus*, was found in alpacas (Rashid et al., unpublished data). Given that very little is known about the epidemiology and diagnosis of GINs of SACs and reliable keys for the identification of L3s of some nematodes (such as *C. mentulatus*) that infect alpacas and llamas remain undescribed, we modified two existing semi-quantitative MT-PCR assays for the GINs of sheep [15, 16] and cattle [16] to accurately detect and differentiate the common GINs, including *C*. *mentulatus*, *Cooperia* spp., *Haemonchus* spp., *Oesophagostomum* spp., *O. ostertagi*, *T. circumcincta* and *Trichostrongylus* spp. in the faeces of alpacas.

## Methods

### Faecal samples and DNA extraction

A total of 35 alpaca faecal samples were available from a previous study (Rashid et al., unpulished data). Following the processing of fresh faecal samples for the faecal egg counts (FECs) of GINs in alpacas by employing the McMaster technique [18], 5 ml of the suspension containing the saturated sugar solution form each sample was drawn and transferred to a 50 ml Falcon tube to extract eggs of GINs as previously described [19]. The washed eggs in each sample were transferred into a microcentrifuge tube and stored at -20 °C until further use. Following thawing, a 250 μl of the concentrated eggs was used to extract and isolate DNA using Powersoil® DNA isolation kit (MO BIO Laboratories, Inc., West Carlsbad, CA, USA) as per manufacturer’s recommended protocol.

### Multiplexed-tandem PCR

We modified the two MT-PCR assays, originally developed for the identification of GINs of cattle [16], and sheep [14, 15] (AusDiagnostics Pty. Ltd., Mascot, New South Wales, Australia) to accurately detect and differentiate the seven common GINs of alpacas, including *C*. *mentulatus*, *Cooperia* spp., *Haemonchus* spp., *Oesophagostomum* spp., *O. ostertagi*, *T. circumcincta* and *Trichostrongylus* spp*.* As *C. mentulatus* is one of the most important GINs in alpacas (Rashid et al., unpublished data), we included it in the alpaca-specific MT-PCR assay by designing new primers, targeting the second internal transcribed spacer (ITS2).

The MT-PCR assay was performed using the *High*-Plex 24 system with the MT-Assay Setup Software for the first round of PCR and the 96-well MT-Analyzer and the MT Analysis Software (Cat. No. 9150, AusDiagnostics) for the nested PCR. Nematode specific primer pairs targeting the ITS2 [Step 1 tubes for nematodes (8-well), Cat. No. 78150S, AusDiagnostics] were used for the primary amplification. Internal specific primers to the ITS2 regions of *C*. *mentulatus*, *Cooperia* spp., *Haemonchus* spp., *Oesophagostomum* spp., *O. ostertagi*, *T. circumcincta* and *Trichostrongylus* spp. (Alpaca Nematodes MP96 8-well, Cat. No. 78150E, AusDiagnostics) amplified ITS2 regions of ~ 90 to 110 bp during the second phase of the assay.

For primary amplification, 5 μl of genomic DNA from test sample or 5 μl of water (negative control) were dispensed into 0.2 ml PCR strips, and placed into a 24-well thermocycling block in the *High*-Plex 24 system (AusDiagnostics).PCR cycling conditions were 15 cycles of 95 °C for 10 s, 60 °C for 20 s, and 72 °C for 20 s. Following the first round of PCR, the secondary amplification and the melting curve analysis were performed in 96-well MT-Analyzer using the MT Analysis Software (AusDiagnostics). A sample was noted as positive using (i) the ‘auto-call function’ of the MT Analysis Software (AusDiagnostics); (ii) if the amplicon produced a single melting curve that was within 1.5 °C of the expected melting temperature; (iii) the height of the peak was higher than 0.2 dF/dT (where dF/dT is the derivative of fluorescence over temperature); and (iv) the peak width was ≤ 3.5 °C. Cycle threshold (Ct) values for each nematode per sample were determined by comparing with the data obtained from the internal spike control (a tube containing a primer pair and 10,000 copies of a synthetic oligonucleotide template in each run). MT-PCR amplicons were randomly selected for sequencing to verify the target nematodes.

The analytical sensitivity was determined using known positive (assessed by amplifying the ITS2 region from each individual worm using a conventional PCR) samples (*C. mentulatus*, *Cooperia oncophora*, *Haemonchus contortus*, *Oesophagostomum venulosum*, *O. ostertagi*, *T. circumcincta* and *Trichostrongylus colubriformis*). The analytical specificity of the assay was assessed by testing known positive samples (*C. mentulatus*, *Chabertia ovina*, *Cooperia curticei*, *C. oncophora*, *C. surnabada*, *H. contortus*, *H. placei*, *Oesophagostomum radiatum*, *O. columbianum*, *C. venulosum*, *Nematodirus spathiger*, *N. filicollis*, *O. ostertagi*, *T. circumcincta, Trichostrongylus vitrinus*, *T. colubriformis*, *T. rugatus* and *T. axei*) by amplifying the ITS2 region from each individual worm using a conventional PCR. Repeatability of the assay for the detection of the expected nematode genera/species and among different runs were also assessed, and the coefficient of variation (CV) was estimated using the program Microsoft Excel (2016) to determine the repeatability.

### Comparison of the MT-PCR data and morphological examination of adult worms

Conventionally, data on the detection of GINs using molecular methods are compared with those of LC [16, 20]. However, we used the morphological identification of adult female and male worms from the same animals because such data are more accurate and specific, and were incidentally available to us from a previous study (Rashid et al., unpublished data). The nematode genera/species found using MT-PCR in the faeces of the 35 alpacas were compared with the results of morphological examination of adult worms collected from the third compartment of the stomach and the small intestine from the same animals to assess the concordance of identification of nematode genera/species. Adult worms were collected from 1/10th of contents of the third compartment of the stomach and the small intestine and then identified based on spicule morphology of male worms (*n* = 15) from each alpaca, using keys described for those which infect small and large ruminants.

### Statistical analysis

The morphological identification of adult worms and MT-PCR datasets were imported into R statistical package for agreement calculations. Adult worms as well as MT-PCR data were converted into binary data based on the presence or absence of nematode genera/species. Frequency table (2 × 2) was constructed for genera/species of GINs and the level of agreement using Kappa values was calculated using *epiR* package [21]. Kappa measures the proportion of agreement and its values are used to compare results of different tests for one set of samples. Kappa has a range from -1 to 1. A benchmark can be used arbitrarily to interpret Kappa values as 0: poor agreement; 0–0.20: slight agreement; 0.21–0.40: fair agreement; 0.41–0.60: moderate agreement; 0.61–0.80: substantial agreement; ≥ 0.81: almost perfect agreement [22].

## Results and discussion

**Fig. 1:**
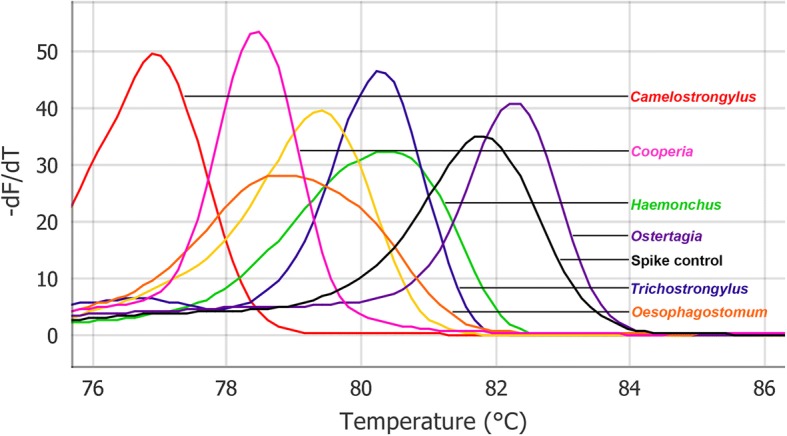
High-resolution melting curve analysis of the gastrointestinal nematodes of alpacas and spike control using the multiplexed-tandem polymerase chain reaction assay

Results revealed that every primer pair used (*n* = 7), successfully amplified the ITS2 region of the target GINs of alpacas (Fig. 1). The specificity of MT-PCR amplicons was verified by DNA sequencing, and no amplification was observed for other nematodes tested. Repeatability of the MT-PCR assay revealed that the seven GINs of alpacas were always correctly assigned (CV of 0%). Based on the peak HRM temperature analyses, each GIN of alpacas was produced as a single and distinct melt curve, and nematodes *C*. *mentulatus*, *Cooperia* spp., *Haemonchus* spp., *Oesophagostomum* spp., *O. ostertagi, T. circumcincta* and *Trichostrongylus* spp. had mean HRM temperatures of 77.0 ± 1.5 °C, 78.4 = 1.5 °C, 80.5 = 1.5 °C, 78.9 = 1.5 °C, 82.3 = 1.5 °C, 79.4 = 1.5 °C and 80.3 = 1.5 °C, respectively (see Fig. 1).

Subsequently, the alpaca faecal samples (*n* = 35) known to be positive for nematode eggs (determined by FECs) were used to validate the modified MT-PCR assay. All 35 samples were test-positive using MT-PCR for at least one nematode genus/species (Table 1). Furthermore, all the seven nematode genera/species (i.e. *C*. *mentulatus*, *Cooperia* spp., *Haemonchus* spp., *Oesophagostomum* spp., *O. ostertagi*, *T. circumcincta* and *Trichostrongylus* spp.) were detected. *Haemonchus* spp. was detected in most of samples (94.29%; 33/35) followed by *Trichostrongylus* spp. (68.57%; 24/35), *C*. *mentulatus* (57.14%; 20/35), *O. ostertagi* (31.43%; 11/35), *Cooperia* spp. (28.57%; 10/35), *Oesophagostomum* spp. (8.57%; 3/35) and *T. circumcincta* (2.86%; 1/35) (Table 1). Mixed infections with two or more nematode genera/species were more common (82.86%; 29/35) than single infections (17.14%; 6/35).

**Table 1:** Agreement (%) between the identification of gastrointestinal nematodes of alpacas using the MT-PCR assay and morphological identification of adult worms

Genera/Species	No. of samples	No. of samples identified by morphological examination (%)	No. of samples identified by MT-PCR (%)	Agreement (%)	Kappa value
*Camelostrongylus mentulatus*	35	22 (62.85)	20 (57.14)	65.71	0.29
*Haemonchus* spp.	35	30 (85.71)	33 (94.29)	85.71	0.22
*Cooperia* spp.	35	18 (51.43)	10 (28.57)	37.14	-0.24
*Teladorsagia circumcincta*	35	1 (2.86)	1 (2.86)	94.23	-0.03
*Trichostrongylus* spp.	35	15 (42.86)	24 (68.57)	38.29	-0.25
*Oesophagostomum* spp.	35	–	3 (8.57)	–	–
*Ostertagia* spp.	35	–	11 (31.43)	–	–

Highest agreement between morphological identification of adult worms and MT-PCR results was observed for *T. circumcincta* (94.23%) followed by *Haemonchus* spp. (85.71%) and *C. mentulatus* (65.71%) (Table 1). Based on Kappa statistic, a fair agreement was found between results of the MT-PCR assay and the morphological identification of worms such as *Haemonchus* spp. and *C. mentulatus*. However, two GINs, *Oesophagostomum* and *Ostertagia* were only detected by the MT-PCR assay (Table 1). Thus, the MT-PCR assay was able to detect more genera/species of alpaca GINs than morphological examination of adult worms.

This is the first study to identify and differentiate GINs using molecular tools in the faeces of SACs. In spite of the development of molecular tools for identifying common GINs in faeces of domestic ruminants in the last two decades [10], LC remains to be the most commonly used method for identifying nematodes at the genus/species level in faeces of domestic ruminants, including alpacas and llamas [2, 23]. However, this technique is not only time and labour-intensive but it also lacks sensitivity and specificity [10]. Furthermore, the taxonomic keys for the identification of L3s of uncommon species of GINs (such as *C. mentulatus*) in SACs remain undescribed which lead to ‘false’ diagnosis of GINs. Contrarily, molecular techniques involving the amplification of nucleic acids which allows minute amounts of target template utilizing specific markers, are effective for the specific identification of GINs [24]. These methods offer accurate, reliable, specific and sensitive tools to traditional approaches such as LC that can not only offer better diagnostic tools of GINs [25]. A range of molecular methods have been developed to detect GINs using DNA isolated from embryonated nematodes eggs or larvae from faeces of ruminants [14–16, 20, 26, 27]. However, MT-PCR offers advantages over most of commonly used molecular tools as it is semi-automated, more sensitive (using nested-PCR) in detecting low infection levels and utilizes genus/species specific primers, thereby allowing simultaneous detection of multiple pathogens in one sample [11].

In this study, we compared the results of MT-PCR with those of morphological identification of adult female and male worms that were available from the same animals whose faecal samples were tested using MT-PCR. Morphological examination of adult worms is considered as a ‘gold standard’ technique in the identification of GINs as this involves the examination of adult male and female worms which provides an accurate and rapid diagnosis of worms. However, LC is laborious and it could be non-specific as it relies on the morphological features of L3s of nematodes developed *in vitro* which can vary depending on temperature and relative humidity during faecal culture, resulting in a bias when assigning larvae to nematode genera/species [28]. Furthermore, significant differences in the protocols of LC often limit direct comparisons of results between or among laboratories [28, 29] whereas the examination of adult worms does not have such issues. Such differences between the identification of GINs based on the examination of adult worms and L3s should be interpreted carefully as adult worms are collected after necropsy while LC is performed based on the collection of faeces of animals using a non-invasive method. However, the use of a ‘gold standard’ test such as the identification of adult worms is critical in the validation of new tests such as the MT-PCR assay developed in this study.

Although a good agreement between morphological identification of adult worms and MT-PCR results were found for *T. circumcincta*, *Haemonchus* spp. and *C. mentulatus* (see Table 1), we found discrepancies between the results of the examination of adult worms and MT-PCR for *Oesophagostomum* spp. and *O. ostertagi* as these were only detected by the MT-PCR assay. This difference between two methods could be attributed to the inherent drawback with the routine total worm count technique which does not involve a meticulous collection of worms (such as *Oesophagostomum* spp.) that occur in the large intestine. Contrarily, molecular tools involving PCRs such as MT-PCR can detect the DNA from even low number of nematode eggs passed in faces of domestic ruminants [24]. Therefore, MT-PCR assay could detect more nematode genera/species of alpacas than morphological examination of adult worms. This difference of sensitivity between the two methods could also be due to the variation in the relative abundance of adult worms collected from gastrointestinal tracts of alpacas as aliquots (rather than complete contents) of stomach and intestinal contents were examined to collect and identify worms during total worm counts. Previously, Roeber et al. [15] also found that the MT-PCR was more sensitive in the detection and differentiation of GINs of sheep. However, these authors compared the results of MT-PCR with those of LC as opposed to identification of adult worms herein.

## Conclusions

The MT-PCR assay established and validated herein, is a rapid, sensitive and effective molecular diagnostic tool to detect and differentiate seven common nematode genera/species of alpacas and llamas. This assay can be used as a substitute to larval culture to identify common nematodes in the faeces of alpacas and llamas.

## Abbreviations

FEC: Faecal egg count
GINs: Gastrointestinal nematodes
ITS2: Second internal transcribed spacer of nuclear ribosomal DNA
LC: Larval culture
MT-PCR: Multiplexed-tandem polymerase chain reaction
SACs: South American camelids
